# Expressions of VEGF-A and VEGFR-2 in placentae from GDM pregnancies

**DOI:** 10.1186/s12958-016-0191-8

**Published:** 2016-09-20

**Authors:** Qian Meng, Li Shao, Xiucui Luo, Yingping Mu, Wen Xu, Li Gao, Haoqin Xu, Yugui Cui

**Affiliations:** 1 Department of Obstetrics, Lianyungang Maternity and Child Health Care Hospital, Lianyungang, China; 2 The State Key Laboratory of Reproductive Medicine, Center of Clinical Reproductive Medicine, First Affiliated Hospital of Nanjing Medical University, 300 Guangzhou Road, Nanjing, 210029 China; 3 Jiangsu Institute of Planned Parenthood Research, 277 Fenghuang Xijie, Nanjing, 210036 China

**Keywords:** GDM, Placenta, Vascular endothelial growth factor, Receptors, Fetal development

## Abstract

**Background:**

Gestational diabetes mellitus (GDM) is one of the most common medical complications of pregnancy, and has important health implications for mother and child. Changes in the fetoplacental vessels may predict those in the vasculature of the developing fetus, as these have been implicated in the pathogenesis of human GDM. This study aimed to determine the differences in the localization and expression level of VEGFA and VEGFR2 between placentas of women with GDM and placentas of normal pregnancies, which is the first step in elucidating the possible roles of VEGFA and VEGFR2 in the altered uteroplacental function resulting from maternal hyperglycaemia and ultimately in the manifestation of GDM.

**Methods:**

The expressions of VEGFA and VEGFR2 mRNA and protein in 20 samples from each group were analyzed by real-time PCR, immunohistochemistry and Western blot. The placental blood barrier and angiogenesis were observed by the transmission electron microscopy (TEM) in10 GDM samples and ten controls.

**Results:**

The expression levels of VEGFA and VEGFR2 mRNA and protein were significantly decreased in the GDM group (*P* < 0.05 or 0.01). Immunohistochemical analysis showed the reduced expression of VEGFA and VEGFR2 protein in GDM-affected placental tissues, and the degenerative alterations of the terminal villi vascular.

**Conclusion:**

The expressions of VEGFA and VEGFR-2 mRNAs and protein were reduced in GDM-affected placental tissues, suggesting that maternal GDM affects the pathophysiological function of placentas.

**Electronic supplementary material:**

The online version of this article (doi:10.1186/s12958-016-0191-8) contains supplementary material, which is available to authorized users.

## Background

Gestational diabetes mellitus (GDM) is defined as any degree of glucose intolerance with onset or first recognition during pregnancy, regardless of whether or not diabetes persists after pregnancy [1–3]. Pregnancy is a diabetogenic state characterized by impaired insulin sensitivity, especially in the second trimester. The major factors contributing to GDM are the placental hormones, such as human placental lactogen, progesterone, cortisol, growth hormone and prolactin. These hormones cause the decreased phosphorylation of insulin receptor substrate-1 (IRS-1), resulting in profound insulin resistance [4–6]. As reported previously, IRS-1 decreases 50–60 % of insulin sensitivity and 50 % of beta cell function in a normal pregnancy [7], and to maintain euglycemia, the pancreas should compensate by increasing insulin secretion by 2–2.5 times. However, beta cell function deteriorates in GDM, particularly during the first phase of insulin secretion.

Gestational diabetes mellitus (GDM) is one of the most common medical complications of pregnancy, and has important health implications for mother and child. Mothers with GDM have an excess of hypertensive disorders during pregnancy, recurrence of gestational diabetes [8], post-partum diabetes and cardiovascular disease thereafter [9–11]. Pregnancies complicated by GDM are associated with several adverse outcomes in the offspring including macrosomia and a longer-term risk of the development of obesity and type 2 diabetes [12].

Fetoplacental vessels are found in chorionic villi bathed in maternal blood, and this close proximity permits efficient exchange of solutes and gases between the maternal and fetal circulations without intermingling of the two. The arrangement allows the development, growth, and remodeling of fetoplacental vessels to be matched to the fetal need while rendering them vulnerable to changes on both the maternal and fetal sides of the placenta. Any pathological alterations in maternal hemodynamics; maternal blood properties (such as hypoxia or hyperglycemia); or growth factors such as vascular endothelial growth factors (VEGFs), soluble fms-like tyrosine kinase-1 (sFlt1), soluble endoglin (sEng) and inflammatory mediators may directly influence the growth, maintenance, and function of fetoplacental vessels. Furthermore, changes in the fetoplacental vessels may predict those in the vasculature of the developing fetus, as these have been implicated in the pathogenesis of human GDM.

VEGF is a homodimeric disulfide-linked glycoprotein involved in both angiogenesis (growth of new blood vessels from existing ones) and vasculogenesis (de novo formation of blood vessels) [13, 14]. VEGF family members include VEGFA, (PlGF, VEGFB, VEGFC, and VEGFD) and two members that are not expressed in mammals (VEGFE, which is expressed in viruses, and VEGFF, which is found in snake venom) [15, 16]. VEGFs initiate cellular responses by interacting with tyrosine kinase receptors on the cell surface. Tyrosine kinase receptors are a specific type of protein kinase receptor that functions by phosphorylating the substrate to stimulate cellular responses. The VEGF receptor (VEGFR) consists of an extracellular domain, a single transmembrane spanning region, and an intracellular component containing a tyrosine kinase domain. There are three main VEGF-R subtypes: VEGFR1 (Flt-1), VEGFR2 (KDR/Flk-1), and VEGFR3 (Flt-4). VEGFA binds to VEGFR1 and VEGFR2; VEGFB, to VEGFR1; and VEGFC and VEGFD, to VEGFR2 and VEGFR3 [17]. The soluble form of VEGFR1 (sFlt1 or sVEGFR1) is a splice variant lacking the transmembrane and cytoplasmic domains. Therefore, it circulates and acts as a potent VEGF and PlGF antagonist by preventing them from reacting with their endogenous receptors [18, 19].

This study aimed to determine the differences in the localization and expression level of VEGFA and VEGFR2 between placentas of women with GDM and placentas of normal pregnancies, which is the first step in elucidating the possible roles of VEGFA and VEGFR2 in the altered uteroplacental function resulting from maternal hyperglycaemia and ultimately in the manifestation of GDM.

## Methods

### Subjects

Women with a history of pregestational diabetes and those with a non-singleton index pregnancy were excluded. A 75-g oral glucose tolerance test (OGTT) was performed with plasma glucose measurement fasting and at 1 and 2 h for women at 24–28 weeks of gestation who were not previously diagnosed with overt diabetes. The OGTT should be performed in the morning after an overnight fast of at least 8 h. The diagnosis of GDM is made when any of the following plasma glucose values are exceeded, based on the American Diabetes Association [20]: (1) fasting ≥ 5.1 mmol/l (92 mg/dl), but <7.0 mmol(126 mg/dl); (2) 1 h ≥ 10.0 mmol/l (180 mg/dl); (3) 2 h ≥ 8.5 mmol/l (153 mg/dl). After GDM was diagnosed, those GDM women were asked diet control to meet the satisfying range of fasting blood glucose (3.3 to 5.6 mmol/L). These patients who were consistently not following the strict management of GDM, without medical therapy initiated. Fourteen patients from this group (*n* = 20) were obese [BMI > 28, BMI = Body weight (kg)/Body surface area^2^ (m^2^)] and four were hypertensive. The clinical data on maternal age and weight, number of gestational weeks, mode of delivery, BMI and weight of placenta were summarized in Table 1. Twenty women with normal pregnancies matched with GDM women for number of gestational weeks, maternal age and mode of delivery were recruited as the control group (*n* = 20). Women in each group provided their placental samples at delivery with the signed informed consent. The study procedure was approved by the ethics committee of the First Affiliated Hospital of Nanjing Medical University (2013-SR-018, 2013) Additional file 1: Table S1.

**Table 1:** Clinical characteristics of GDM women and the control group. Results were expressed as the mean ± SD or number (percentage)

Characteristics	Control group (*n* = 20)	GDM group (*n* = 20)	*P* value
Gestation, weeks	39.41 ± 0.96	39.00 ± 0.90	0.34^a^
Maternal age, years	28.4 ± 5.5	31.4 ± 6.7	0.13^a^
Maternal BMI, kg/m^2^	24.27 ± 2.18	28.23 ± 2.94	0.02^a*^
Mode of delivery	Cesarean	Cesarean	-
Sex of newborn			0.53^b^
Male	9(45)	12(60)	
Female	11(55)	8(40)	
Birth weight of newborn, g	3621 ± 380	3744 ± 537	0.29^a^
Placental weights, g	734 ± 31.5	752 ± 33.2	0.11^a^

*BMI* body weight (kg)/body surface area^2^ (m^2^). ^a^Student’s t test.; ^b^ *x* ^2^ test. *P < 0.05

### Placental sample collection

In this study, all the subjects underwent delivery by cesarean section due to various reasons such as anxiety and tension, a burning desire of cesarean section and excessive afraid of pain,, other than those related to pathological procedures, to avoid the potential affects of the delivery procedure or other pathological factors on the expression of VEGF system. The placental specimens were weighed and performed on the basis of obstetric indications. The tissue specimen was dissected from the placental subchorial zone corresponding to the umbilical cord insertion (approximately 5 cm away from the site of cord insertion), while avoiding areas of infarction and hematomas. Placentas with abnormal umbilical cord insertions such as velamentous cord insertion were excluded from the analysis. Tissue fragments from the placenta were cut longitudinally from the maternal side to the fetal side. Placental tissues were divided into three parts, maternal, middle, and fetal, as described by Sood et al. [21]. The middle part, consisting of homogeneous villous tissues, was collected and placed in ice-cold phosphate-buffered saline (PBS), cleaned of blood, and immediately cut into four 1 cm × 1 cm × 1 cm fragments, which were frozen and stored at –80 °C for RNA analysis.

### TEM examination

For the ultrastructural examination, ten women in each group were randomly selected to provide their placental samples at delivery (Additional file 1: Table S1). Until delivery, eight GDM women (8/10) kept in the A1 class (fasting glucose less than 5.8 mmol/L, postprandial blood glucose less than 6.7 mmol/L) after good diet control. Two GDM women (2/10) were classed as A2 (fasting blood glucose higher than or equal to 5.8 mmol/L, postprandial blood glucose higher than or equal to 6.7 mmol/L) because of their poor diet control. The levels of fasting blood glucose and HbA1c in those GDM women with good diet control in the last weeks of gestation were kept in the range of 5.1 to 5.6 mmol/L. However, the level of fasting blood glucose (6.1 and 11.3 mmol/L) and the level of HbA1c (10.2 and 10.3 mmol/L) in two GDM women with poor diet control were higher than the satisfactory criteria. Women with normal pregnancies matched with GDM women for number of gestational weeks, maternal age and mode of delivery were recruited as control (Additional file 1: Table S1). Three villous tissue blocks per placental sample were prepared for the ultrastructural examination using TEM. The samples were fixed in 2.5 % glutaraldehyde in cacodylate buffer, and stored in cacodylate buffer with 0.05 M saccharose (pH 7.2) at 4 °C until processing. The villous tissues were then post-fixed in 1 % OsO4 for 2 h at 4 °C, routinely processed in a graded series of acetone, then infiltrated with acetone-araldite and embedded in araldite. For orientation, semithin sections (thickness, 1 μm) were stained with thionine. Ultrathin sections (thickness, 80 nm) were treated (double contrast) with uranyl acetate (25 min) and 8 % lead nitrate (5 min) and then systematically examined in a JEM-1010 electron microscope (JEOL Ltd).

Terminal villi were evaluated with respect to the placental blood barrier (thickness of the vasculo-syncytial membrane, thickness of the syncytiotrophoblast (ST) basal membrane (BM), and thickness of the endothelial BM). For each tissue block, five microscopic fields were randomly selected and systematically investigated at 5000×, 12,000×, and 25,000× magnification for quantitative analysis. Thus, a total of 15 random fields were recorded and analyzed per placenta to minimize individual differences. The following three measurements were performed for each field: thickness of the vasculo-syncytial membrane from the intervillous space to the fetal vessels, perpendicular to the BM, at 5000× magnification; microvillous density per 10 μm of length at 12 000× magnification; and thickness of the ST or endothelial BM was measured at 25,000× magnification. Images were analyzed using the TEM Image Platform (Olympus) to perform random measurements. The two operators who carried out the microscopic analyses were blinded to the placental group until the end of the study.

### Immunohistochemistry

Twenty tissue blocks in each group were sectioned and fixed to slides with the placenta and stained together. VEGF-A immunostaining: Slides were de-waxed, rehydrated, antigen retrieved and bathed in hydrogen peroxide for 30 mins. Blocking was done with Normal Donkey Serum (NDS). Rabbit anti-VEGFA antibody (VEGF (ab105219) (1:50) (500 μg/ml) (Abcam) and DAR-555 (1:1000) were used as primary and secondary antibody respectively.

VEGFR2 Immunostaining: Slides were de-waxed, rehydrated, antigen retrieved and bathed in hydrogen peroxide for 30 mins. NDS was employed to block non-specific antibody binding. Rabbit anti-VEGFR2 antibody (55B11) (1:200) (100 μg/ml) (Cell Signaling) and DAR-555 (1:1000) were used as primary and secondary antibody respectively.

### RNA extraction and cDNA preparation

Total RNA was extracted from twenty placenta tissues from each group using Trizol Reagent (Invitrogen, Carlsbad, CA). RNA was treated with DNase using TURBO DNA-free kit (Ambion, Austin, TX), and purified with an RNeasy mini kit (Qiagen Inc., Valencia, CA). Spectrophotometric analysis and gel electrophoresis were used to determine the yield, purity, and integrity, and to ensure the lack of genomic DNA contamination in the samples. cDNA was prepared from total RNA using a high-capacity cDNA reverse transcriptase kit (Applied Biosystems, Foster City, CA).

First-strand synthesis was performed on 2 μg of total RNA using Superscript II/III ribonuclease H-reverse transcriptase (Invitrogen, Australia).

### Real-time PCR

The mRNA expression of *VEGF-A* and *VEGFR-2* in the placental samples obtained from the GDM and control pregnancies were quantified in an ABI Prism 7700 (Perkin-Elmer/Applied Biosystems) using prevalidated Assays on Demand (consisting of a 20× mix of unlabeled *HLX1*-PCR primers and FAM-dye labeled TaqMan MGB probe; *HLX1* Assays on Demand, catalog no. 4331182; Applied Biosystems). Gene expression was quantified as the second step in a two-step RT-PCR protocol according to the manufacturer’s instructions. In brief, the 20-μL PCR reaction mix contained TaqMan Universal PCR master mix, 1× Assays on Demand gene expression assay mix, and 1 μL of placental cDNA. The sample was amplified for 40 cycles, including a denaturation step at 95 °C for 15 s and an annealing/extension step at 60 °C for 30 s. The oligonucleotide primers used for the amplification of the gene VEGF-A and VEGFR-2 were as follows: VEGF-A forward primer, 5′-TGCGGATCAAACCTCACCAA-3′ and VEGF-A reverse primer, 5′-TGTCACATACGC TCCAGGACTT-3′; VEGFR-2 forward primer, 5′-GTGTCAGAATCCCTGCGAAGTA-3′ and VEGFR-2 reverse primer, 5′-GAAATGGGATTGGTAAGGATGA-3′. Actin was used as a housekeeping gene. The primers were designed using Primer Express 1.5 software (Applied Biosystems). A mixed sample was used as the sample of quality control (QC). In every assay, the QC sample was added to two wells in the same 96-well plate. The average Ct of actin was calculated and normalized to test the variation within a assay. The relative value of target gene in the QC sample was 2^(Ct2-Ct1)^, which was used to correct the variation between assays. The relative quantitation of VEGF-A and VEGFR-2 expression normalized to actin was calculated according to the 2^–ΔΔT^ method of Livak and Schmittgen [22] using a term control as a calibrator (ABI Prism 7700 sequence detection system, User Bulletin no. 2, 2001).

### Western immunoblotting

Twenty samples from each group were tested as following protocol. Total protein was extracted from 20 mg of snap-frozen placental tissue in 200 μL of RIPA lysis buffer containing 50 mmol/L of Tris-HCl (pH 7.4), 1 % Triton X-100, 1 % sodium deoxycholate, 150 mmol/L NaCl, 0.1 % sodium dodecyl sulfate (SDS), sodium orthovanadate, sodium fluoride, ethylenediaminetetraacetic acid (EDTA), and leupeptin using an Ultra-Turrax (Ika-Labortechnik). The homogenized samples were centrifuged at 10,000 *g* for 5 min at 4 °C to sediment any insoluble material. The protein concentration of the supernatant was determined using an Enhanced BCA protein assay kit (Beyotime, China) with bovine serum albumin (Sigma Aldrich, Australia) as the standard. Approximately 25 μg of protein per lane was fractionated using 10 % SDS-polyacrylamide gel electrophoresis (PAGE). The proteins were electrophoretically transferred to nitrocellulose membranes and blocked with 5 % nonfat milk in Tris-buffered saline (pH 7.4). Purified rabbit polyclonal VEGFA (1/200; ab105219; VEGFA-121, 165, and 189; Abcam) and rabbit monoclonal VEGFR2 (1/1000; 55B11; Cell Signaling) were used as the primary antibodies. Antibody binding was visualized using peroxidase-conjugated rabbit anti-mouse (Zymed, Mulgrave, Australia) secondary antibody, and analyzed by autoradiography using Fluorchem™ 5500 (Amersham Biotech, Shanghai, China).

The total protein in each well was detected by Coomassie blue staining to ensure constant protein load. A mixed sample as the QC sample was added to two wells in every plate from different assay. Tublin was used as a housekeeping protein. The densitometry of tublin was determined, and then the relative value of target protein in the QC sample was normalized. The variation within a assay was evaluated by two relative values, while the variation between assays was also corrected by the mean relative value. The expressions of VEGFA and VEGFR2 protein were semiquantitatively determined using Scanning Densitometry (Image Quant, Australia).

### Statistical analysis

Data were expressed as the mean and standard deviation (SD). Statistical analyses were performed using SPSS software (Statistical Analysis System, version 17.0 for Windows). Statistical differences between the two groups were analyzed using Student’s t test for those data in the normal distribution. Statistical significance was set at *p* < 0.05.

## Results

### Summary of the clinical features

As shown in Table 1, the GDM group (*n* = 20) had a significantly higher mean BMI than the control group (28.23 ± 2.94 kg/m2 versus 24.27 ± 2.18 kg/m2, *P* = 0.02). Other parameters such as gestation, maternal age, mode of delivery, sex and birth weight of the newborn, and placental weight did not significantly differ between the groups.

### TEM analysis

The placenta barrier was composed of ST, endothelium and the space between them. ST was a continuous syncytial layer with multiple nucleus and numerous apical microvilli, while CT had a single large nucleu and scattered distributed beneath the syncytium so that could not be seen in all the visual fields, which is analogous in both groups. BM of ST was a continuous, uniform, and thin basal lamina where collagen fibrils occasionally deposited, which was same to the BM of fetal endothelium. The stroma was the connective tissue core of the chorionic villous space separating ST from the capillary endothelium BM, and contained different kinds of stromal cells and bundles of collagen fibrils.

**Fig. 1:**
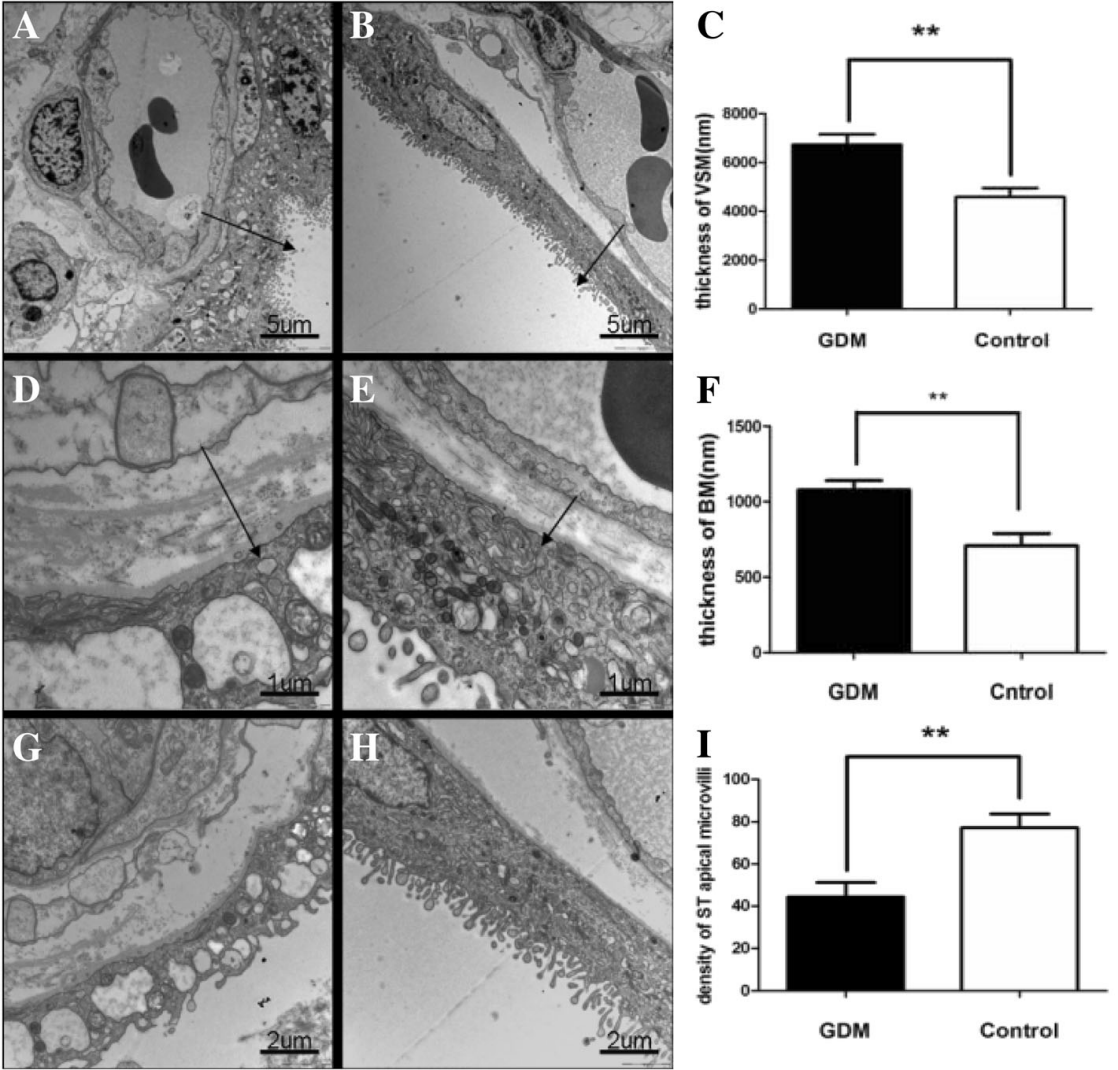
Comparison of semiquantitative parameters of placental ultrastructures between the GDM group (*n* = 20) and the control group (*n* = 20). **a**-**b**. The terminal villi of GDM (**a**) and control (**b**) placenta. **c** Placental barrier thickness (*arrow*) of the GDM group (6746.15 ± 1270.22 nm, *n* =10) was significantly thicker than that of the control group (4591.34 ± 1178.60 nm, *n* = 10; *P* < 0.05), Bar = 5um. **d**-**e** The terminal villi of GDM (**d**) and control (**e**) placenta. **f**. The BM of ST in the GDM group (1077.49 ± 194.39 nm nm, *n* = 10) was thicker than that in the control group (707.54 ± 256.56 nm, *n* = 10; *P* < 0.05), Bar = 1um. **g**-**h** The density of ST apical microvilli in GDM (**g**) and control (**h**) placenta. **i** The density of ST apical microvilli in the GDM group (44.36 ± 21.95 per 10um, *n* = 10) was significantly lower than that in the control group (77.13 ± 20.82 per 10um, *n* =10; *P* < 0.05), and even microvilli-free in some areas, Bar =2um

The placental blood barrier and angiogenesis were specially observed by TEM analysis. The vasculo-syncytial membrane (VSM) and BM of ST were significantly higher (*P <* 0.05) in the GDM group (6746.15 ± 1270.22 nm and 1077.49 ± 194.39 nm, respectively; Fig. 1a and d; *n* = 10) than those in the control group (4591.34 ± 1178.60 nm and 707.54 ± 256.56 nm, respectively; Fig. 1b and e; *n* = 10). The density of ST apical microvilli per unit surface area in the GDM group (44.36 ± 21.95 per 10 μm) was significantly lower than that in the control group (77.13 ± 20.82 per 10 μm; *P <* 0.05), as shown in Fig. 1g–i.

### Localization and distribution of VEGFA and VEGFR2

**Fig. 2:**
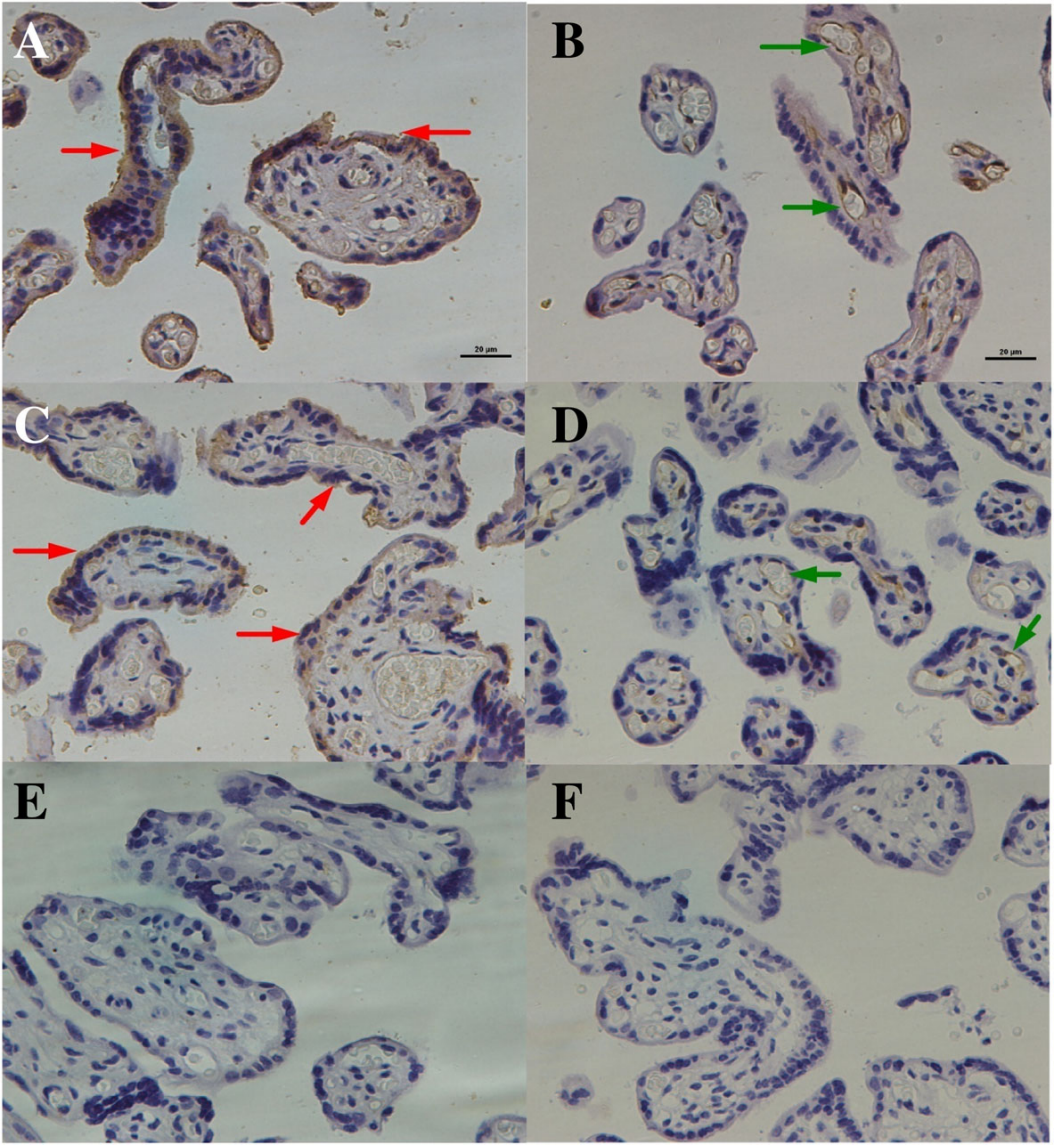
Immunohistochemical staining to study the cellular localization of VEGFA and VEGFR2 proteins in the GDM and control placentas. Representative controls (**a**, **c** and **e**) and GDM placentas (**b**, **d** and **f**) were stained with rabbit monoclonal antibodies to VEGFA (**a** and **b**) and VEGFR2(**c** and **d**). **e** and **f** Omission of the primary antibody as control. Syncytiotrophoblasts (*red arrows*) and fetal vessels (*green arrows*). Scale bars: 20 μm

IHC was used to investigate the cellular distribution of VEGFA and VEGFR2 proteins in GDM-affected (*n* = 20) and control (*n* = 20) placental tissues. Figure 2a and c showed the representative images of VEGFA immunoreactivity in the ST layer of term control tissues (Fig. 2a) and term GDM tissues (Fig. 2c). Qualitative assessment of the immunoreactivity revealed that the VEGFA protein level was lower in the placentas of the GDM group than in those of the control group but with no discernable difference in protein localization. Figure 2b and d showed the localization and distribution of the VEGFR2 protein in the placental tissues. Qualitative analysis revealed weaker staining in the endothelial cells of fetal vessels in the placentas of the GDM group (Fig. 2d) than that in the control group (Fig. 2b), but no discernable difference in the localization of VEGFR2 was noted.

### VEGFA and VEGFR2 mRNA expression

**Fig. 3:**
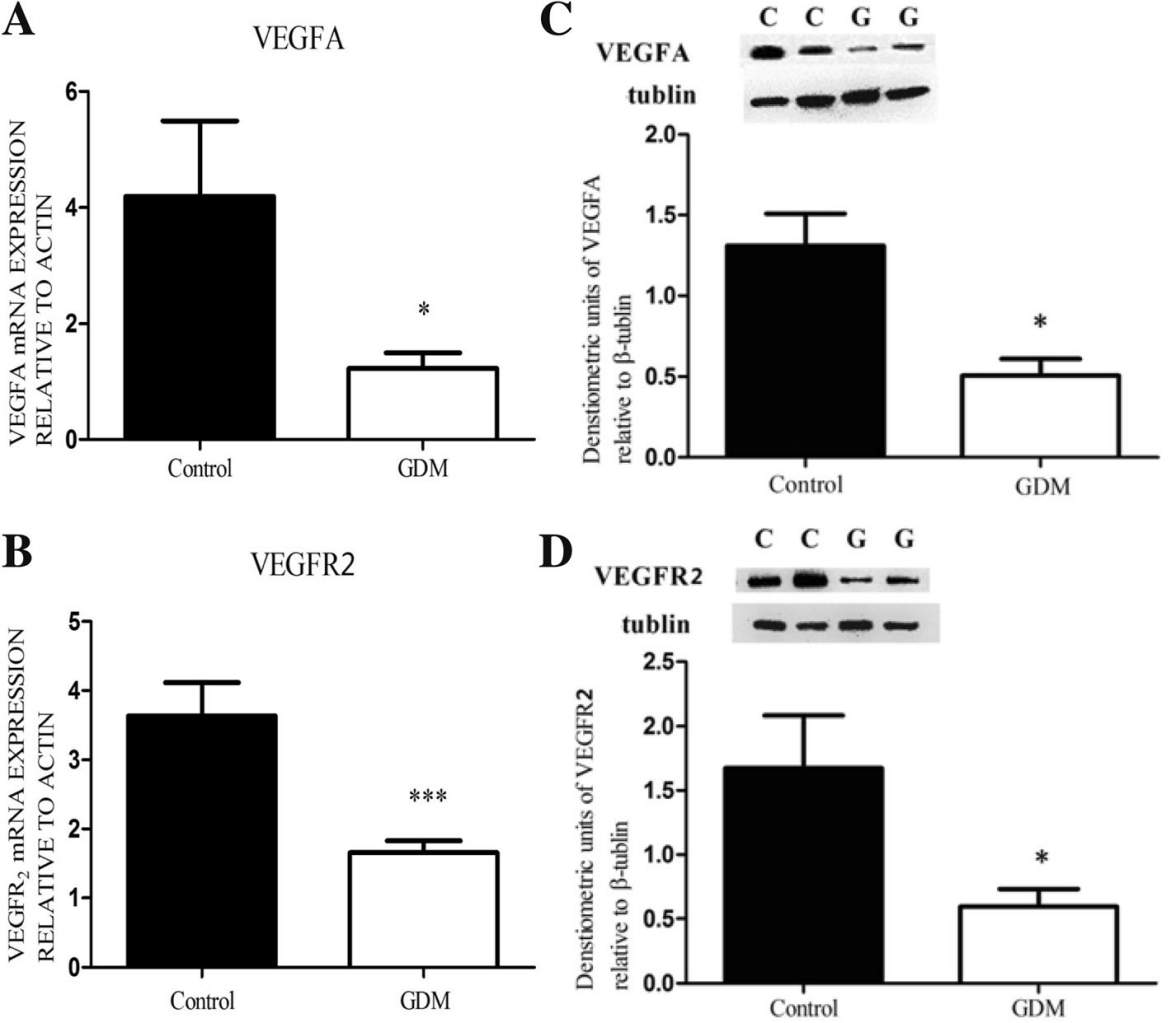
Expressions of VEGFA and VEGFR2 mRNAs and proteins in GDM (*n* = 20) and control (*n* = 20) placental tissues by real-time PCR and Western immunoblotting. Relative quantification of *VEGFA* (**a**) and *VEGFR2* (**b**) mRNA expression normalized to the expression of *ACTIN* in all the samples. Data were analyzed according to the 2^–ΔΔCT^ method. A representative immunoblots for VEGFA (**c**) and VEGFR2 (**d**) were shown. Immumoblots representing β-tubulin protein (*middle panels*) showed the loading equally total protein (25 μg). Semi-quantitative analyses of VEGFA and VEGFR2 immunoreactive proteins were performed relative to β-tubulin (*bottom panels*). The Y-axis represents the expression levels of *VEGFA* and *VEGFR2* related to *actin* or *tubulin*. *P < 0.05, **P < 0.01, Student’s t test

Figure 3 illustrated the expressions of *VEGFA* and *VEGFR2* mRNAs. Relative to the level of the *Actin* housekeeping gene, the *VEGFA* mRNA level was significantly decreased in the GDM group (*n* = 20) when compared with the control group (*n* = 20) (1.23 ± 0.53 versus 4.19 ± 2.61, *P* < 0.05; Fig. 3a). Similarly, the relative expression of *VEGFR2* mRNA was significantly reduced in the GDM group when compared with control group (1.66 ± 0.33 versus 3.64 ± 0.95, *P* < 0.01; Fig. 3b).

### Protein expression of VEGFA and VEGFR2

Figure 3 also showed the representative immunoblots of VEGFA and VEGFR2 proteins in the GDM-affected (*n* = 20) and control placentas (*n* = 20). Representative immunoblots of the 51-kDa housekeeping protein β-tubulin was used to illustrate the protein load of all samples. The 27-kDa immunoreactive VEGFA protein was observed in both groups. Semi-quantitative densitometry showed the decreased expression of VEGFA protein in the GDM group when compared with the control group (0.51 ± 0.21 versus 1.31 ± 0.40, *P* < 0.05; Fig. 3c). Figure 3d showed a 210-kDa protein of VEGFR2 in the GDM and control samples. Densitometry also showed the decreased VEGFR2 protein expression in the GDM group when compared with the control group (0.59 ± 0.49 versus 1.60 ± 1.47, *P* < 0.05).

## Discussion

The primary origin of fetal derangements in gestational diabetic mothers is fetal hyperglycaemia resulting from maternal hyperglycaemia. This hyperglycaemia may result in the metabolic and hormonal change in the fetus. Once the fetal pancreas commences to produce and secrete insulin in the second trimester [23], fetal hyperglycaemia will result in fetal hyperinsulinemia and stimulation of fetal metabolism. Lower fetal oxygen content associated by higher lactate concentrations may certainly reflect enhanced fetal metabolism as a result of hyperglycaemia and hyperinsulinemia [24]. Consequently, the improvement of fetal oxygen demands will aggravate the chronic fetal hypoxia [25]. The hyperglycemia of GDM develops during pregnancy and clinically manifests only in the late second trimester and thus may have an impact on placental processes occurring in later stages of pregnancy, such as angiogenesis and microvascular remodeling. VEGF/VEGFR-2, which was selected as representative factors related to the placental blood barrier and angiogenesis in this study, is important in promoting vascular endothelial cell growth and in increasing the number of vessels and capillaries [22], thereby ensuring the adequate supply of nutrients to the fetus, through classical feedback mechanism. We found that the expressions of VEGFA and VEGFR2 was significantly lower in the placentas of women with GDM although we did not show the relationship between above expressions and the increased glucose levels or abnormal levels in their OGTTs due to the limited sample size. There is consistent evidence that the concentration of VEGF in GDM maternal and cord plasma was decreased [26]. VEGF mRNA expression and protein production are oxygen-dependent, which are known to be up-regulated by hypoxia [27]. Just because of the strong proangiogenic potency of hypoxia through regulating multiple steps of vascular growth [28], chronic fetal hypoxia as the consequence of maternal diabetes may thus stimulate placental vasculogenesis and angiogenesis by increasing the growth factors expression in the placenta and fetus. Parallel to its regulation by oxygen, placental VEGF is at a high level in the first trimester when oxygen levels are low and decline thereafter towards term of gestation [29–31], moreover the extent of hyperglycemia other than hypoxia may also contribute and modify its effect [32]. Thus, the condition of hyperglycemia induces a state of mild and persistent ischemia and hypoxia with subsequent increase of hypercapillarization. In contrast, high blood glucose levels especially poorly controlled in GDM trigger severe hypoxia/ischemia, with inhibition of binding VEGF/VEGFR-2 and consequent reduction of hypercapillarization, and therefore fetal hypoxia as a result of maternal diabetes does not stimulate the expression of VEGF in the third trimester [33]. The complex process of villous development and maturity might be also influenced by the maternal and fetal oxidative and other angiogenetic milieu, which will result in endothelial dysfunction and oxidative stress [34]. As the placenta is one of major sources of VEGF during pregnancy, our findings suggested that the reduced expression of VEGF in placenta in the GDM group may contribute to the pathogenic vascular defects which could be observed on the histological examination. Two other studies have also reported that the placental expression and fetal cord levels in GDM are significantly lower than normal [32,35].

These data were confirmed by immunolocalization of VEGF and VEGFR-2. The distribution of VEGF was observed in the placentas of women with GDM, and no VEGF was detected in the placental cellular compartments, which suggested a decrease in VEGF production. VEGF was always detected in the in the ST layer of term control tissues and term GDM tissues [32], in women with gestational diabetes there was a significant decrease in VEGF expression profile when compared to normoglycemic women. The target cells for VEGF in the chorionic villi were determined based on the immunolocalization of VEGFR-2, which expression was particularly low in the placental capillary endothelial cells of gestational diabetes, in contrast to normoglycemic women. The binding between VEGF and VEGFR-2 triggers a signaling pathway that negatively regulates angiogenesis.

Our finding showed that distinct alternations of ultrastructure in GDM placenta comparing with control placenta. The significant differences in ultrastructure may be summarized as the thickening of the vasculo-syncytial membrane (VSM) and BM of ST, a decreased number of ST apical microvilli per unit surface area in the GDM group. These changes related to the reduced VEGFA and VEGFR2 expressions may have adversely affected the transport efficiency of the placental vasculature by the decreased transport of oxygen, nutrients, and waste across the placenta [36]. To explore the comparable and inchoate changes in the VEGFA and VEGFR2 expression and placental ultrastructure, we excluded those cases with adverse pregnancy outcomes or pathological placental tissuess in this study. Our results suggested that this effect may be a response to the placental hypoxia induced by hyperglycemia, which might have potential affect on the placental and fetal development and growth during pregnancy.

## Conclusion

This observational study revealed the reduced expressions of VEGFA and VEGFR2 in the placental tissues obtained from women with GDM. It should be noticed for both clinicians and GDM women that the reduced VEGF/ VEGFR2 may affect the placental blood barrier and angiogenesis, and consequent placental function, in the pregnancy with GDM.

## Additional file

Additional file 1: Table S1. The detailed clinical information of 10 cases for TEM exam from each group. (DOCX 15 kb)

## Abbreviations

BM: Basal membrane
BMI: Body mass index
GDM: Gestational diabetes mellitus
HbA1c: Glycosylated hemoglobin A1c
IRS-1: Insulin receptor substrate-1
NDS: Normal donkey serum
OGTT: Oral glucose tolerance test
PBS: Phosphate-buffered saline
sEng: soluble endoglin
sFlt1: soluble fms-like tyrosine kinase-1
ST: Syncytiotrophoblast
TEM: Transmission electron microscopy
VEGF: Vascular endothelial growth factor
VEGFA: Vascular endothelial growth factor A
VEGFR: Vascular endothelial growth factor receptor
VEGFR2: Vascular endothelial growth factor receptor-2
VSM: Vasculo-syncytial membrane
