# Supplementary material for: Expressions of VEGF-A and VEGFR-2 in placentae from GDM pregnancies
Source: Reprod Biol Endocrinol. 2016 Sep 20;14:61. doi: 10.1186/s12958-016-0191-8 (PMC5029036; doi:10.1186/s12958-016-0191-8)
Supplement: Additional file 1: Table S1. — The detailed clinical information of 10 cases for TEM exam from each group. (DOCX 15 kb) [file 12958_2016_191_MOESM1_ESM.docx]

**Supplemental Table 1.** **The detailed clinical information of 10 cases for TEM exam from each group .** The diagnosis of GDM is made when any of the following plasma glucose values are exceeded: (1) fasting≥5.1 mmol/l (92 mg/dl), but <7.0mmol(126mg/dl); (2) 1 hour≥10.0 mmol/l (180 mg/dl); (3) 2 hours≥8.5 mmol/l (153 mg/dl). After GDM was diagnosed, those GDM women were asked diet control to meet the satisfying range of fasting blood glucose (3.3 to 5.6 mmol/L). Women with normal pregnancies matched with GDM women for number of gestational weeks, maternal age and mode of delivery were recruited as the control group. For the ultrastructural examination, ten women in each group were randomly selected to provide their placental samples at delivery. Until delivery, eight GDM women (8/10) kept in the A1 class (fasting glucose less than 5.8 mmol/L, postprandial blood glucose less than 6.7 mmol/L) after good diet control. Two GDM women (2/10) were classed as A2 (fasting blood glucose higher than or equal to 5.8 mmol/L, postprandial blood glucose higher than or equal to 6.7 mmol/L) because of their poor diet control. The levels of fasting blood glucose and HbA1c in those GDM women with good diet control in the last weeks of gestation were kept in the range of 5.1 to 5.6 mmol/L. However, the level of fasting blood glucose (6.1 and 11.3 mmol/L) and the level of HbA1c (10.2 and 10.3 mmol/L) in two GDM women with poor diet control were higher than the satisfactory criteria.

| **Cases** | **Age**  **(yr)** | **BMI**  **(kg/m^2^)** | **Gravidity** | **Previous**  **parity** | **Gestational wk**  **at delivery** | **Mode of**  **delivery** | **Sex of**  **baby** | **Birth**  **weight (g)** |
| --- | --- | --- | --- | --- | --- | --- | --- | --- |
| GDM 1 | 29 | 28.36 | 1 | 0 | 40.3 | Caesarean | Male | 3800 |
| GDM 2 | 31 | 29.61 | 4 | 0 | 39.2 | Caesarean | Female | 5000 |
| GDM 3 | 27 | 28.20 | 1 | 0 | 38.0 | Caesarean | Male | 2750 |
| GDM 4 | 30 | 29.21 | 1 | 0 | 39.7 | Caesarean | Male | 3850 |
| GDM 5 | 28 | 27.44 | 1 | 0 | 38.1 | Caesarean | Female | 3100 |
| GDM 6 | 36 | 28.45 | 3 | 2 | 39.7 | Caesarean | Female | 3750 |
| GDM 7 | 31 | 25.10 | 2 | 0 | 38.0 | Caesarean | Male | 3250 |
| GDM 8 | 27 | 29.10 | 1 | 0 | 39.1 | Caesarean | Female | 4150 |
| GDM 9 | 24 | 26.94 | 3 | 2 | 38.0 | Caesarean | Male | 3250 |
| GDM 10 | 41 | 29.91 | 3 | 1 | 39.9 | Caesarean | Female | 4000 |
| Cont 1 | 27 | 24.32 | 1 | 0 | 40.7 | Caesarean | Female | 3500 |
| Cont 2 | 29 | 26.04 | 2 | 0 | 39.3 | Caesarean | Female | 4100 |
| Cont 3 | 26 | 21.60 | 1 | 0 | 40.0 | Caesarean | Male | 3250 |
| Cont 4 | 28 | 23.59 | 5 | 1 | 38.9 | Caesarean | Female | 3600 |
| Cont 5 | 28 | 24.41 | 1 | 0 | 38.4 | Caesarean | Male | 3850 |
| Cont 6 | 28 | 24.57 | 1 | 0 | 38.0 | Caesarean | Male | 4000 |
| Cont 7 | 23 | 22.85 | 2 | 0 | 40.4 | Caesarean | Female | 3450 |
| Cont 8 | 23 | 25.44 | 3 | 1 | 40.4 | Caesarean | Female | 4000 |
| Cont 9 | 35 | 24.15 | 2 | 0 | 38.4 | Caesarean | Female | 3000 |
| Cont 10 | 27 | 26.64 | 1 | 0 | 39.6 | Caesarean | Male | 4150 |
